# 

**Published:** 1901-05

**Authors:** 


### PERSONALS.

Dr. James W. Sallade, of Pottsville, has removed to Auburn, Pa. Dr. T. H. McCarthy succeeds him at his former location.

Dr. James A. Waugh, of Pittsburg, met with a misfortune by fire at his hospital on Forbes Street, early in April, gutting the entire first floor.

Dr. W. S. Hinchcliff, of Grand Tower, Ill., is the owner of several high-class stallions, and conducts a breeding establishment in connection with his practice.

Dr. G. L. Salisbury, Jr., of Wickford, R. I., is a trainer of trotters and pacers, and frequently acts as considering judge in races.

The veterinary inspectors for the Philadelphia open-air horse-show to be held from May 27 to June 1, 1901, are Drs. C. J. Marshall, W. H. Ridge, and Charles A. Dohan.

Dr. N. B. Rhodes maintains an able sanitarium in connection with his practice at Tampa, Florida.

Dr. N. A. Boucher, of Minneapolis, Minn., has removed to Salina, Kan.

Dr. William Herbert Lowe, of Paterson, N. J., was the victim of a serious attack of inflammatory rheumatism in April, and was confined to his bed for several weeks.

Dr. H. F. Kauffman, of Klingerstown, Pa., has removed to Ashland, Pa., where he will continue the practice of his profession.

Dr. W. Horace Hoskins, of Philadelphia, was a visitor to Atlantic City in March.

Dr. H. D. Gill, of New York City, continues to attract marked attention along the speedway with his fast pacemakers. It is said that most drivers fight shy of him in brushing.

Dr. Hal C. Simpson, Assistant State Veterinarian of Iowa, has just resumed practice after an extended absence. Dr. Simpson was in the English service and accompanied a transport of horses to South Africa, and was veterinarian in the United States Government's transport service to Manila, P. I. Dr. Simpson had the misfortune to be shipwrecked on the coast of Hayti, in May last, when 580 mules were lost out of 1450.

Dr. P. K. Jones, of Pittsburg, Pa., is about to give his entire attention to a stock-farm practice at Wellsburg, Va., where he will establish very palatial kennels. Dr. Jones was a visitor to Philadelphia, New York, and Boston.

